# Exploiting Resistive Matrix Technology to Build a Stretchable Sensorised Sock for Gait Analysis in Daily Life

**DOI:** 10.3390/s22051761

**Published:** 2022-02-24

**Authors:** Nicola Carbonaro, Lucia Arcarisi, Carlotta Marinai, Marco Laurino, Francesco Di Rienzo, Carlo Vallati, Alessandro Tognetti

**Affiliations:** 1 Department of Information Engineering, University of Pisa, 56124 Pisa, Italy; lu.arcarisi@gmail.com (L.A.); marinai.carlotta@gmail.com (C.M.); francesco.dirienzo@phd.unipi.it (F.D.R.); carlo.vallati@unipi.it (C.V.); alessandro.tognetti@unipi.it (A.T.); 2 Research Center E. Piaggio, University of Pisa, 56124 Pisa, Italy; 3 National Research Council, Institute of Clinical Physiology, 56124 Pisa, Italy; laurino@ifc.cnr.it

**Keywords:** gait analysis, textile sensors, daily life monitoring, smart textiles

## Abstract

We describe the development and preliminary evaluation of an innovative low-cost wearable device for gait analysis. We have developed a sensorized sock equipped with 32 piezoresistive textile-based sensors integrated in the heel and metatarsal areas for the detection of signals associated with the contact pressures generated during walking phases. To build the sock, we applied a sensing patch on a commercially available sock. The sensing patch is a stretchable circuit based on the resistive matrix method, in which conductive stripes, based on conductive inks, are coupled with piezoresistive fabrics to form sensing elements. In our sensorized sock, we introduced many relevant improvements to overcome the limitations of the classical resistive matrix method. We preliminary evaluated the sensorized sock on five healthy subjects by performing a total of 80 walking tasks at different speeds for a known distance. Comparison of step count and step-to-step frequency versus reference measurements showed a high correlation between the estimated measure and the real one.

## 1. Introduction

The way we walk consists of repeated step cycles, which include a predefined sequence of gait phases (heel-strike, stance, heel-off, and swing). Both temporal (e.g., cadence, support time, step time, single support time, and double support time) and spatial (e.g., step length, stride length) characteristics of gait are important for evaluating a disease aand for defining and optimizing its treatment [1,2,3]. Gait analysis exploits the extraction of both temporal and spatial parameters of gait for the evaluation and treatment of patients suffering from a large number of pathologies that affect walking [4].

The pathologies related to walking alterations are many and of different natures, ranging from neurodegenerative to chronic pathologies. Neurodegenerative diseases are a major cause of disability among older people. It is estimated that a very high number of patients suffer from Parkinson’s or Alzheimer’s, with numbers that will rapidly increase in the near future [5,6]. Many studies have shown that gait alterations are one of the first observable effects in patients suffering from neurodegenerative diseases [7,8]. For example, people with Parkinson’s disease have difficulty starting or finishing steps (akinesia [9]), walk slower than normal [10], and walk with shorter steps than normal [11]. With regard to chronic pathologies, for example, chronic obtrusive pulmonary disease (COPD) patients have alterations in gait patterns such as reduced stride length, increased time in double support, reduced cadence, and greater walking variability [12]. The speed of gait slows as COPD severity increases and correlates with clinical symptoms, lung function, and quality of life scores [13]. Walking speed (i.e., obtained by combining spatial and temporal parameters) is a predictor of readmission risk in patients admitted for acute COPD exacerbation [14]. Regardless of the pathology, walking speed is considered a reliable clinical outcome, so much to be considered the *sixth vital sign* [15]. In clinical practice, the loss of mobility associated with a reduced gait speed is normally assessed through standard clinical tests such as the Six-Minute Walk distance (6MWD), which measures the distance an individual is able to walk over a total of six minutes on a hard, flat surface [16,17]. Standard tests, such as 6MWD, give important information on patient average speed, but they do not evaluate how the natural gait speed evolves in time and they are not able to extract other gait characteristics. On the other hand, standard technologies used to perform gait analysis, such as podobarometric platforms [18] or optical systems [19], are obtrusive, not usable in daily context, expensive, and require the supervision of expert operators.

Given this premise, it is clear that there is a strong need to develop sensorized systems that allow for a pervasive analysis of the walking activity of patients at risk during normal conditions of daily life. In addition to detecting changes associated with certain pathologies, the massive use of these devices would allow for the generation of new knowledge obtainable through the retrospective exploration of walking data associated with the clinical outcomes of the subjects examined. To achieve these objectives, it is necessary to develop acquisition systems that are non-invasive, low-cost, and easily usable by users. In this study, we describe the development and preliminary evaluation of an innovative low-cost wearable device for gait analysis. We have developed a sensorized sock equipped with a high number of piezoresistive textile-based sensors integrated in the heel and metatarsal areas for the detection of signals associated with the contact pressures generated during the walking phases. Among the main events that characterize the walking cycle of a subject, we can select the moment in which the heel touches the ground and the moment in which the toe comes off the ground. The first event is identified as Heel Strike (HS), while the second as Toe Off (TO). A gait cycle is therefore defined as that time interval between two consecutive HS events of the same foot. Furthermore, a gait cycle can be divided into two phases: the stance phase, the period in which the foot touches the ground, identified as the time interval between an HS and a TO of the same foot; and the swing phase, the period in which the foot is in the air. The swing phase begins immediately after the stance period, and the separation of the two periods is identified by the TO event. In this work, we studied the ability of the sensorized sock to estimate some simple parameters of the subject’s walk, such as number of steps and frequency of steps, starting from the detection of HS and TO events. We decided to develop a sensorized sock because sock is a garment that everyone wears every day, and it is not limited by fashion, like shoes. We integrated the textile-based sensors by applying a sensing patch on a commercial sock. We designed the sensing patch as a stretchable circuit in which conductive stripes, based on conductive inks, are coupled with piezoresistive fabrics to form the sensing elements. The principle of the sensing patch—the core component of our sensorized sock—is based on the resistive matrix method. The resistive matrix method employs a piezoresistive layer (middle layer) inserted between two layers (bottom and top layers) with conductive stripes that face the piezoresistive layer. The bottom and top layers are oriented to arrange the conductive stripes in perpendicular directions. By convention, the conductive stripes of the bottom and top layers are referred to as rows and columns. Rows and columns have the function of picking up the signal from specific areas of the piezoresistive layer. The reading electronics uses a multiplexer strategy by sequentially selecting all columns and measuring the corresponding resistances seen from each row. In this way, a matrix is created with a number of sensors equal to the product of the number of rows and the number of columns, and each sensing element is represented by the piezoresistive material of the intersection between a row and a column. Cheng et al. demonstrated the resistive matrix method on textile substrates in [20], where the resistance of each sensing element changes when a normal pressure is applied. In our previous works, we adapted the resistive matrix method for the development of a textile-based piezoresitive array applied in a sensing mattress for sleep analysis and physiological signal detection [21,22]. In [23], we exploited the resistive matrix method for the development of a preliminary version of the sensorized sock. The great advantages of the resistive matrix method are that the connecting wires to the reading electronics can be applied on the periphery of the matrix and that no electrical contact is created on the sensing areas, thus increasing mechanical robustness and strongly decreasing the number of electrical contacts (e.g., the reduction factor for a square resistive matrix is $k=\frac{\sqrt{n}}{2\;n}$, where n is a perfect square indicating the number of sensing areas). The main disadvantage is the complexity of the signal routing due to the positions of the connections placed on the two opposite layers of the matrix. For this reason, the method is mainly applied in large area solutions such as those reported in [20,21,22], while it finds less application in personal wearable devices. In addition, the existing textile solutions based on the resistive matrix method use rigid fabrics (both for the piezoresitive layer and the conductive tracks on the top and bottom layers) and, as far as we know, there are no stretchable solutions that use the resistive matrix method. Another disadvantage of the classical resistive matrix method is the sensor cross-talk due to the parasitic electrical resistances associated with the surface conductivity of the continuous piezoresistive layer [24]. In fact, as reported by [24], an electrical resistance is formed between adjacent parallel electrodes due to the electrical reclosure provided by the continuous piezoresistive layer. This electrical resistance, commonly called the parasitic resistance, directly affects the reading of a single sensitive element. In our sensorized sock, we introduced many relevant improvements to overcome the limitations of the classical resistive matrix method, and in the current paper, the strategies and method used to build the prototype are explained in detail. In addition, we have developed dedicated algorithms to extract simple walking features such as step count and step-to-step frequency. We tested the sensorised sock on five healthy subjects by performing a total of 80 walking tasks at different speeds for a known distance. Promising results were obtained by comparing the step count and step-to-step frequency versus reference measurements; this showed high correlation between the estimation measure and the real one.

Other sensorized sock solutions can be found in the literature [25,26,27,28]. In general, existing solutions use a limited number of discrete sensors (up to six). Eizentals et al. [25] have developed a smart sock with six pressure sensors built by integrating conductive pathways on the sock. The work of D’Addio et al [26] presents a sensorised sock based on three textile sensors and an accelerometer incorporated into the fabric and connected by a conductive thread. In [27], Langer and colleagues show a smart sock with four pressure sensors based on piezoresistive textile technology. The work of Lin et al. [28] describes a textile solution with a low number of sensors that measures the pressure variation due to walking activity; it should be noted that the prototype is battery-free (powered by RF energy). In all these prototypes, the number of sensors is limited due to the complexity of the sensor connection that has to be created in the detection area. The low number of sensors reduces the spatial resolution, making it difficult to adapt the prototype to different foot sizes and conformations. On the contrary, our solution, based on the adaptation of the resistive matrix method, allows us to monitor the plantar pressures due to walking with sensor arrays characterized by a relatively high spatial resolution. The sensor redundancy makes the precise positioning of the single sensor unnecessary; thus, our solution is robust with respect to the normal structural differences that exist between the feet of different subjects. In future studies, we plan to use the high number of sensitive elements integrated in our sensorized sock to also carry out static analysis of the subject’s posture, thereby evaluating the balance and the support modality of the entire plantar arch. We obtained a high spatial resolution of the sensors through the implementation process that we describe in this work and that modifies the resistive matrix technology, making it compatible with wearable and stretchable devices. Furthermore, none of the aforementioned works [25,26,27,28] has carried out the extraction of simple temporal characteristics of the gait by comparing them with reference systems.

## 2. Materials and Methods

### 2.1. Device Concept

We built the sensorised sock prototype by integrating a stretchable textile circuit (hereinafter referred as the *sensing patch*) with 32 piezoresistive sensors in a commercially available sock. Sensors were applied in the metatarsal and heel regions to detect pressure distribution due to ground reaction forces during walking (i.e., 16 sensors in the heel and 16 sensors in the metatarsus). We chose to integrate the sensors below heel and metatarsus because they are the areas where the body weight is distributed more during walking [29]. The sock selected (PUTUO sock available on Amazon.it) is long, elastic, and has separate fingers. It is made of cotton to ensure breathability and lightness. We chose the design with separate fingers to prevent the user from misplacing the sensors, thus ensuring more robust and repeatable measurements. In addition, the separate fingers increase the absorption of sweat, improving the user comfort and also inhibiting the formation of bacteria. A long sock gives greater mechanical stability and the ability to connect electronics in a simple way. Given the elasticity of the sock and the sensing patch, it is possible to use a single device for different foot sizes (e.g., our sock covers a range from 39 to 45 EU), which represents a great advantage over sensorized insoles that require specific measures. Note that we could have chosen any type of commercial sock that had the necessary characteristics of elasticity and wearability. A dedicated wearable electronic unit detects the signals of the 32 sensors and transmits the measurements to a smartphone via Bluetooth. The electronics are connected to the sensing patch at the back of the calf. The sensorised sock is based on a low-cost production strategy: the total cost of the prototype, including the sock, the sensing patch, and the wearable electronics, is below 100 €, which could be further reduced in a future production phase.

### 2.2. Sensing Patch Design and Sock Fabrication

We designed the sensing patch starting from the concept of the resistive matrix method but introduced many relevant improvements to overcome the limitations described in Section 1.

**Figure 1:**
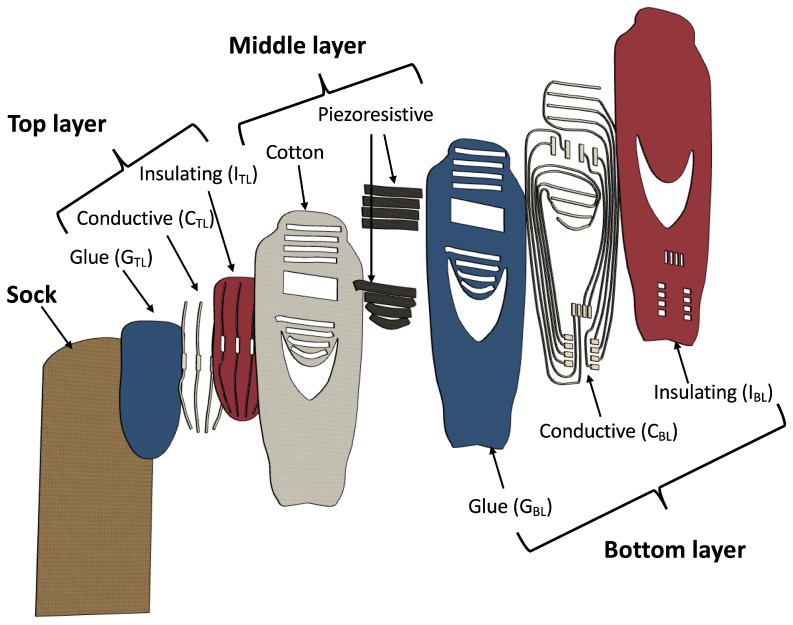
Details of the prototype and building blocks of the top, middle and bottom layers. The top layer consists of a continuous glue layer, a conductive ink pattern, and an insulating ink pattern that is open over the conductive stripes to ensure electrical contact with the piezoresitive element when pressure is applied. The bottom layer consists of a glue pattern, a conductive ink pattern, and an insulating ink pattern. The $G_{BL}$ pattern covers the connection tracks to ensure proper insulation and is open over the row conductors to ensure the electrical contact with the piezoresistive material of the middle layer. The middle layer consists of 8 piezoresistive stripes that are glued on a perforated cotton fabric in correspondence of to heel and metatarsal areas.

Figure 1 shows the structure of the top, middle, and bottom layers and their arrangement with respect to the sock.

**Figure 2:**
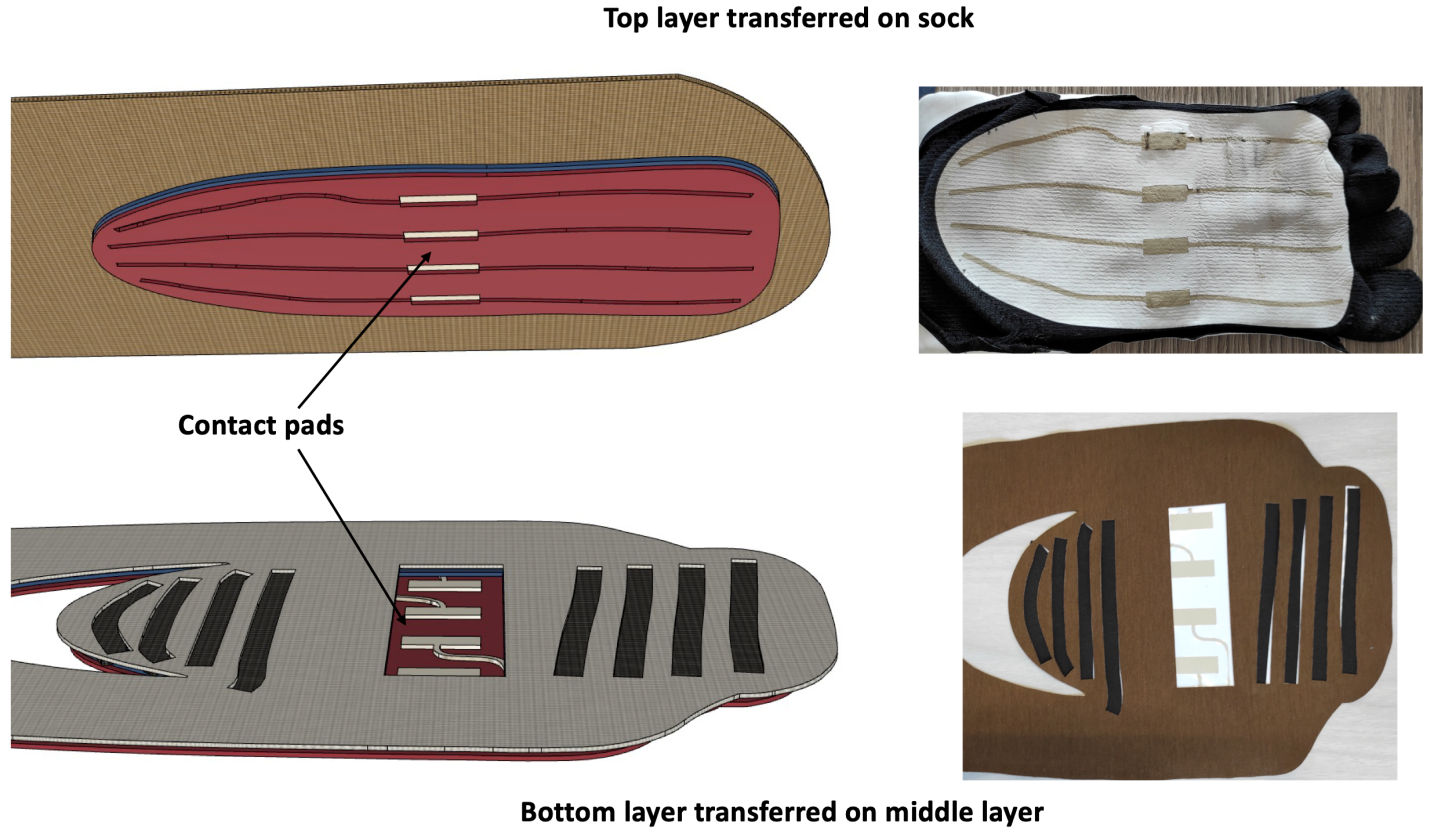
Detail of how we perform the electrical connections of the elements of the upper layer with the lower one. On the left is the representation of the CAD project, and on the right is the detail of the sensorized sock made. The procedure consists of transferring the top layer to the sock and the bottom layer to the middle layer. The union of the bottom and middle layer is then stitched to the sock to form the stretchable sensing patch. In the center of the layers, the contact pads are shown. The contact pads are used to make the electrical connections of the conductive strips of the upper layer with those of the lower layer using a 3M Z-axis conductive tape. In this way, it was possible to locate all electrical contacts of the sensing patch on a single layer, i.e., the lower layer.

We built the top and bottom layers of the sensing patch by employing the print transfer technique. In the print transfer technique, commonly used to apply graphic elements on fabrics, different stratum of inks and a thermoplastic adhesive are screen printed on a sacrificial support. The screen printed structure is then transferred to the fabric using a thermal press thanks to the activation of the thermoplastic adhesive. Once it has returned to room temperature, the sacrificial support is removed by pulling it from one end. Note that the last printed stratum is always the thermoplastic adhesive, which is therefore on the opposite side with respect to the sacrificial stratum. Finally, a curing phase of the transferred sample is carried out to ensure the best electrical performance. We designed the top layer of the sensing patch to obtain four conductive stripes (the columns) that cover the entire length of the foot. The top layer consists of a continuous glue layer ($G_{TL}$ in Figure 1), a conductive ink pattern ($C_{TL}$ in Figure 1), and an insulating ink pattern ($I_{TL}$ in Figure 1). Note that the $I_{TL}$ is open over the conductive stripes to ensure electrical contact with the piezoresitive element when pressure is applied. We designed the bottom layer of the sensing patch to obtain 8 conductive stripes (the rows) that cover the heel and metatarsal areas. The bottom layer also includes the 8 conductive tracks that connect the rows and the columns to the reading electronics. The bottom layer consists of a glue pattern ($G_{BL}$ in Figure 1), a conductive ink pattern ($C_{BL}$ in Figure 1), and an insulating ink pattern ($I_{BL}$ in Figure 1). The $G_{BL}$ pattern covers the connection tracks to ensure proper insulation, while it is open over the row conductors to ensure the electrical contact with the piezoresistive material of the middle layer. We designed the middle layer to obtain 4 piezoresistive stripes in the heel area and 4 piezoresistive stripes in the metatarsal area. The middle layer consists of 8 piezoresistive stripes that are glued on a perforated cotton fabric. We obtained the piezoresistive stripes by cutting the CARBOTEX 03-82 fabric (produced by SEFAR AG, Heiden, Switzerland) with a laser-cutter machine (Trotex 100, Trotec, Concorezzo, Italy). Note that the CARBOTEX fabric is flexible but not elastic. We perforated the cotton fabric with the same laser-cutter machine. We conceived the middle layer structure with the piezoresistive stripes applied on the areas of interest to: (1) obtain elasticity (note the cotton fabric is elastic while the piezoresitive fabric is not stretchable), (2) reduce the parasitic resistances typical of the resistive matrix method (using the stripes we eliminated parasitic resistance only in the column direction), and (3) save material and costs. In addition, the thickness of the cotton acts as a spacer (typical of force sensing resistors) that insulates the piezoresistive stripes from the columns conductors when no pressure is applied on the sock. According to the print transfer technique and as shown in Figure 2, the top layer is transferred to the sock while the bottom layer is transferred to the middle layer. The structure formed by the bottom layer transferred on the middle layer is then sewn onto the sock to form the sensing patch.

Following our design, the top and bottom layers were built by Eptatech (Italy). The layers provided by Eptatech are highly stretchable; they can tolerate deformations up to 15% of their original size thanks to the combination of stretchable dielectric/conductive inks and thermoplastic glue. In particular, the conductive stripes of the top and bottom layers are made of a water-based Silver conductive ink (sheet resistivity: <30 mΩ/sq at 0% elongation). We performed the transfer process using a heat press (180 °C, 15 s, 4 bar) that activates the thermoplastic glue. We peeled-off the sacrificial substrate, and we performed the final curing at 150 °C for 5 min.

A relevant aspect of our design is the four contact pads that we have drawn in the central part of the upper and lower layers (see Figure 2). In the construction phase of the sock, we put in contact the pads of the upper layer with those of the lower layer by using a 3M (Saint Paul, MN, USA) Z-Axis conductive tape (the Z-axis tape provides electrical contact only in the thickness direction and is commonly used to bond two conductive surfaces). With this expedient, it was possible to bring the connections of the upper layer conductive strips back to the lower layer and to locate all the 12 electrical contacts of the sensing patch (4 columns, 4 lines for the metatarsus, and 4 lines for the heel) on the lower layer. Note that all the 12 connections to the electronics—3 blocks of 4 silver pads placed in the ankle area—are on the same layer, which is commonly not the case of the classical resistive matrix method. Concerning the fabrication of the prototype, we have filed an Italian patent application in the name of the University of Pisa.

### 2.3. Wearable Electronics and Mobile Application

**Figure 3:**
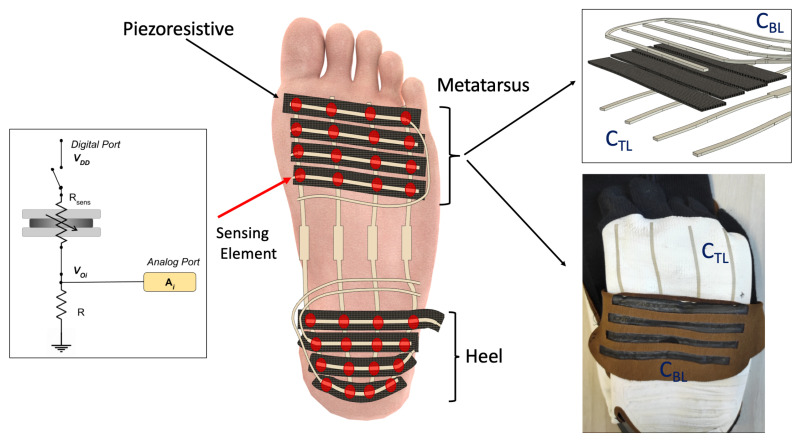
Details of multilayer pressure-sensitive textile matrix. Red circles at the row/columns intersections identify the 32 sensing elements. The three-dimensional representation of the rows, columns, piezoresistive stripes, and their arrangement in the metatarsus area are represented in the inset on the top, while in the inset on the bottom it is possible to see the structures of the columns ($C_{TL}$) and rows ($C_{BL}$) of the sensorized sock made. The rows are black due to the piezoresistive material attached to them. Finally, the inset on the left shows how a single sensing element ($R_{sens}$) is read: one end (column) is attached to a digital port, and the other end (row) is connected to a 10 kΩ resistor and to an analog port of the Arduino Nano 33 BLE Sense board.

According to the description of Section 2.2 and as shown in Figure 3, we have obtained 32 sensing elements divided into two different blocks of 4 × 4 arrays, located in the heel and metatarsus areas, respectively.

Each sensing element behaves like a variable resistor whose electrical resistance decreases once the applied pressure increases. To acquire the raw signals associated with the variation over time of the electrical resistance of the 32 sensing elements, we developed a multi-channel electronic unit able to sequentially activate each sensing area through appropriate management of the sensing patch rows and columns. The electronic unit is based on the Arduino Nano 33 BLE Sense board. As shown in the inset of Figure 3 for the i-th sensing element, we employed a voltage divider scheme, where $R_{sens}$ is the variable resistance to be determined and R is a 10 kΩ pull-down resistor. The resulting $V_{oi}$ is a function of the electrical resistance of the single sensing element. We connected the 4 columns (top layer) to 4 digital channels of the Arduino Nano board and the 8 rows (bottom layer) to the 8 analog channels. To read the resistance value of each sensing element, we sequentially activate the digital channels (switching from GND to VDD) and simultaneously acquire all the 8 analog channels. The Arduino Nano board performs the analog to digital conversion and the transmission of the raw data to a smartphone via Bluetooth. The 32 sensing elements of the patch were sampled at the frequency of 25 Hz. In addition, we acquired the signals of the inertial measurement unit (IMU) integrated in the Arduino board, although we did not use them in this study. The raw sensor data ($V_{oi}$) and IMU values were transmitted through dedicated Bluetooth services. We made 2 sensorized socks, one for the right foot and one for the left foot, and we implemented two different strategies to connect the 32 patch sensing elements to the electronic unit. The first strategy was used in the left sock, in which we designed and integrated a dedicated magnetic connector. To enable the electric connection, we sewed neodymium magnets (40 × 10 × 3 mm) on the sock in correspondence to the silver pads, and we integrated iron rectangles (40 × 15 × 1 mm) in the 3D printed case of the electronic unit. In the right sock, we soldered unipolar cables to to copper foil layers that were glued to the silver pads of the sensing patch using a 3M Z-axis conductive tape.

In addition, we have designed and developed a custom software application to manage and collect all the sensors data. The application, running on an Android smartphone, automatically searches for and connects to the wearable electronics. Once the BLE connection is completed, the wearable electronics begin sending sock sensor data and IMU values to the smartphone. Raw data of the sensing elements and IMU values are displayed in real time via a graphical user interface. In addition, the application locally saves all sensors data, and these can subsequently be exported via email for off-line analysis, also facilitating long-term experiments.

### 2.4. Step Detection and Walking Frequency Estimation

To emphasize the signal variation due to walking or running, we calculated the differential signal (X(t)) between the average signals of metatarsal area ($X_{m}\left(t\right)$) and heel area ($X_{h}\left(t\right)$) as follows:

$$X\left(t\right)=X_{m}\left(t\right)-X_{h}\left(t\right)$$

The mean signals $X_{m}\left(t\right)$ and $X_{h}\left(t\right)$ were estimated by averaging the signals from the sensing elements connected to lines in the metatarsus area and lines in the heel region (see Figure 3), respectively. The differential signal *X*(*t*) was band-pass filtered (Chebyshev-II filter) in the frequency range 0.2–3 Hz, to preserve spectral information of walking or running conditions. Then, the signal was processed using a Savitzky–Golay smoothing filter with a 3nd degree polynomial with 25 neighbours.

Two different algorithms were developed to estimate the step count and the step-to-step frequency, respectively.

To estimate the step count, the local maxima and minima of differential signal *X*(*t*) were extracted. The features of local maxima and minima were (i) a minimum amplitude of 40 mV for maxima and a maximum amplitude of −40 mV for minima and (ii) a minimum temporal distance from other maxima or minima of 100 ms. The step count was estimated as half of the number of zero-crossing of local maxima and minima sequence.

The step-to-step frequency was evaluated as a function of time by splitting the X(t) signal in epochs of 2 s time duration. For each *i*-epoch, *n* moving overlapped windows ($x_{i1}\left(t\right),\ldots x_{ik}\left(t\right)\ldots x_{in}\left(t\right)$) of 5 s duration centered within the *i*-epoch were extracted from X(t), and the overlapping time between the windows was set to 0.1 s. For each $x_{ik}\left(t\right)$, an auto-correlation sequence $R_{ik}\left(\tau \right)$ was estimated as a function of time lag τ with a minimum lag of 0.2 s (step-to-step frequency of 5 Hz) and a maximum lag of 5 s (step-to-step frequency of 2 Hz). Each auto-correlation sequence was normalized by setting its value at zero-lag to one. The maxima of each $R_{ik}\left(\tau \right)$ were estimated by considering at least a difference threshold of 0.4 between each maximum and its surrounding values. For each *i*-epoch of X(t), we obtained a sequence of auto-correlation maximum lags of length *n* (number of overlapped windows). Each lag of the absolute maximum of each auto-correlation sequence $R_{ik}\left(\tau \right)$ was an estimation of the time periodicity of *i*-epoch of X(t). The weighted arithmetic mean of the auto-correlation maximum lag sequence was estimated by considering as weights the auto-correlation peak value of each lag. The step-to-step frequency of each *i*-epoch was calculated as reciprocal of correspondent weighted arithmetic mean. Therefore, we obtained a sequence of step-to-step frequency as a function of time. Finally, the outliers of sequence of step-to-step frequency were removed by Hampel filtering with a sliding window of 10, and a difference of 3 standard deviations at least. The mean step-to-step frequency of each trial was estimated by averaging the step-to-step frequency sequence.

To statistically compare the results between the left and right socks, we pooled the difference between the estimations and references obtained for each subject and test (pooled error vector) for both step count and step-to-step frequency estimations. We excluded from the data pooling only subject 4, because the data collected by subject 4 were highly artefacted and noisy. The pooled error vectors were estimated for left and right socks, and they were compared by using a paired Wilcoxon Signed Rank Test.

### 2.5. Laboratory Tests

**Figure 4:**
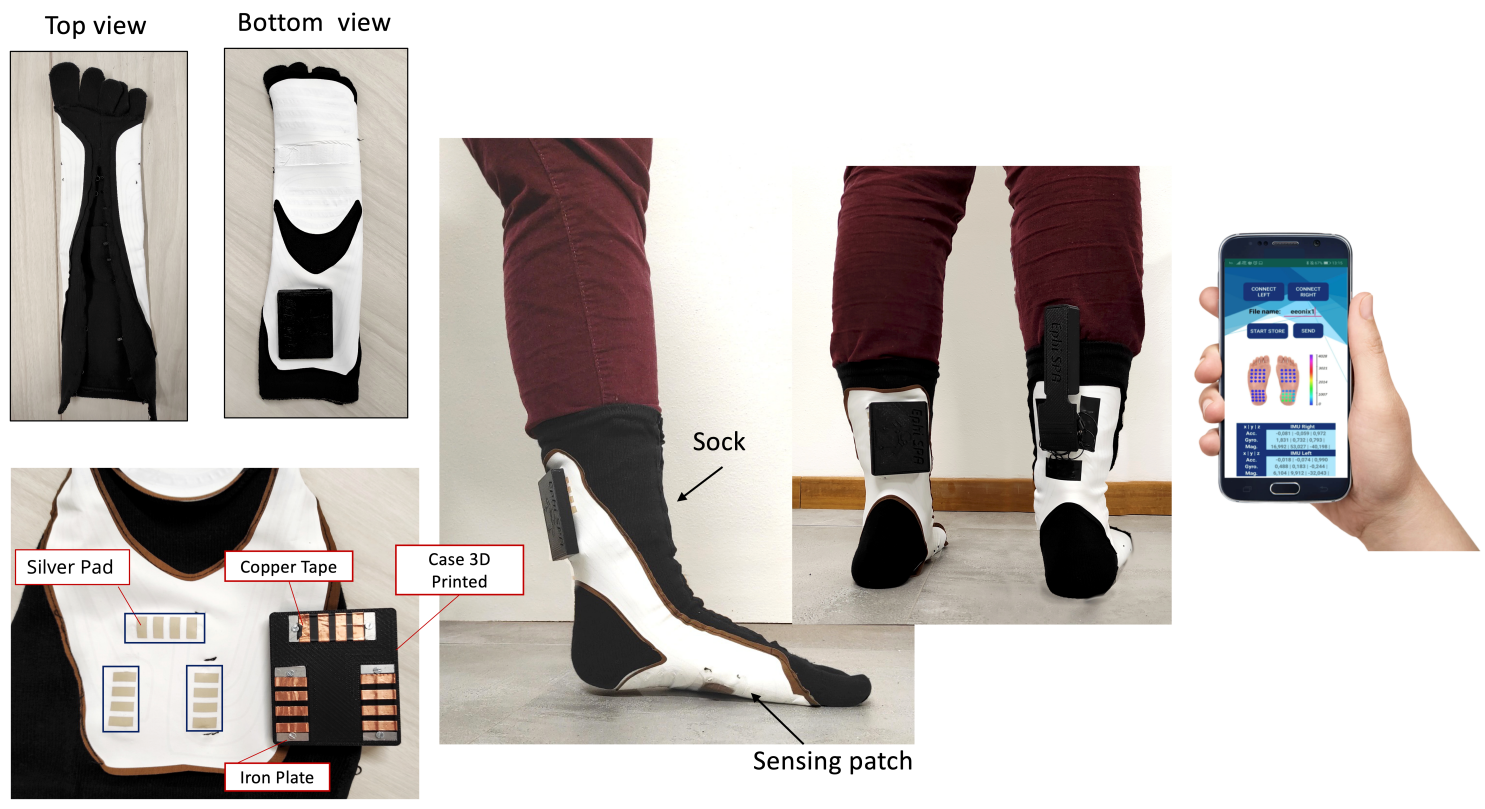
The sensorized sock prototypes. The left side of the figure shows the prototype details, highlighting the location of the 12 electrical connections (3 blocks of 4 silver pads) and the electronic unit with the dedicated magnetic connector. The picture on the right shows the main screen of the developed mobile application.

To demonstrate the ability of our sensorized sock to detect significant parameters of the gait cycle, we carried out walking tests on 5 subjects (five young healthy women with height between 162 cm and 178 cm and weight between 54 kg and 73 kg, for other characteristics refer to Table 1). We asked each subject to walk in a corridor while wearing the two socks (i.e., left foot and right foot, see Figure 4).

During the test, the subject walked for a known distance, 9 m, at different speed (normal, slow, very slow, and fast). We wanted to test the system during free walking, in those conditions that best represent the activities of everyday life. For this reason, we have not foreseen the use of further laboratory equipment that could condition and/or limit the movement of the subject. We used a video camera (RealSense D435 manufactured by Intel) so that we could film the complete test of each subject. Then, we manually analyzed the individual tests by noting the specific information of the subject’s walking activity such as number of steps, time elapsed between one step and the next one, instant of time related to heel strike, and toe-off events. This procedure was developed in order to have a reference measurement to compare the parameters extracted from our sensorized sock obtained by using the algorithms described in Section 2.4. We acquired a total of 80 trials (16 trials for each subject). We processed the data through the algorithm described in Section 2.4 to extract gait parameters such as step count and step frequency and compare the results with the reference measurement.

## 3. Results and Discussion

### 3.1. Prototype and Raw Data

We have realized two sensorized socks and two electronic units, one for the left foot and one for the right foot, and an Android application that allows one to display in real time sensors data and to store locally. The sock chosen, having the shape of the heel and toes, allows the subject to wear it correctly and consequently ensures that the sensors are placed in the desired areas. The sensing patch structure consists of two main layers: the first, the one that integrates the columns (CTL), was directly transferred to the sock in order to follow its elasticity and ability to adapt to the subject’s foot. The second layer, containing the rows and stripes of piezoresistive fabric, is sewn only along the side edges for the entire length of the sock. With this choice of binding the second layer only on the lateral edges, the first layer is free to follow the lengthening of the sock due to the different size of the subject’s foot. The sensing patch has a thickness of only 0.12 mm and a weight of about 3 g. The sensorized sock prototypes, the wearable electronics, and the mobile application are shown in Figure 4.

**Figure 5:**
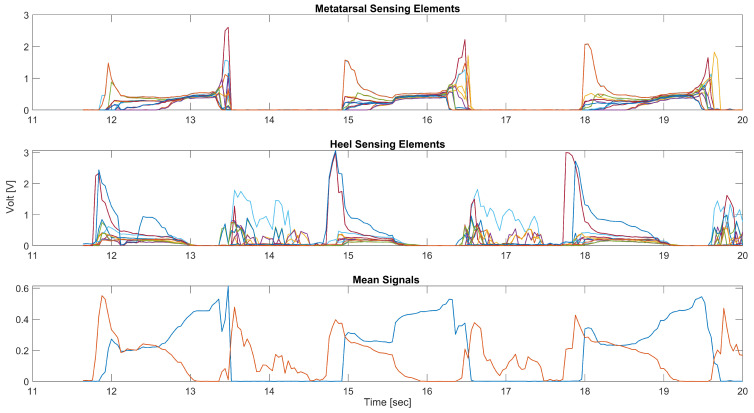
Raw signals acquired during one of the tests. The **top** and **center** figures show, respectively, the signals obtained from the 16 sensors of the metatarsal and heel areas during 3 gait cycles. The **bottom** figure represents the trend of the $X_{m}\left(t\right)$ and $X_{h}\left(t\right)$ signals, calculated as the average of all the signals obtained from the metatarsal and heel areas.

Figure 5 shows the raw signals of the sensorized sock collected from the mobile application during a typical walking task. The 16 signals obtained from the sensing elements located in the metatarsal and heel area, reported in the top and central figures, show the peculiar trend of a gait cycle due to the pressure exerted by the different areas of the foot on the ground. The high number of sensors positioned in the areas of interest, metatarsal, and heel guarantee that a sufficient number of sensors are well positioned to detect fundamental information on the subject’s walk. This is demonstrated by the fact that it is possible to carry out a simple average of all the values of the sensing elements of the metatarsus or heel to determine the events of HS or TO, rather than through more complex signal processing.

**Figure 6:**
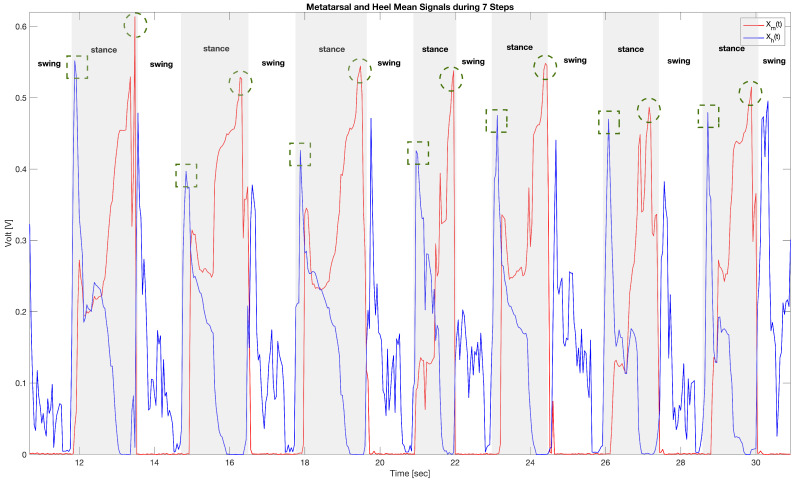
The figure represents the typical trend of the signals acquired during 7 steps. Shaded areas highlight stance intervals respect to swing periods. Moreover, dotted square indicates HS events dashed circle TO events.

In this work, we studied the ability of the sensorized sock to estimate some simple parameters of the subject’s walk, such as number of steps and frequency of steps, starting from the detection of HS and TO events. In fact, looking at the Figure 6, it is possible to identify the HS event as the maximum variation in pressure recorded by the sensitive elements of the heel, that is, the local maximum of the $X_{h}\left(t\right)$ signal. Similarly, the sensorized sock makes it possible to identify the TO event as the maximum pressure variation detected by the sensitive elements of the metatarsal area, i.e., the local maximum of the $X_{m}\left(t\right)$ signal. Furthermore, as shown in Figure 6, the alternation of the stance and swing phases, typical of the common gait cycle, is clearly visible (the stance intervals are highlighted by the shaded areas). Figure 6 shows the signals obtained during a test in which the subject performed 7 steps. At the beginning, the foot is in the swing stage and all sensor values are close to zero. Immediately after, there is a peak in the heel signal, so we are in the HS phase (indicated by the dotted square in Figure 6), which shows how the subject’s weight is fully loaded on the heel. Subsequently, the whole foot is in contact with the ground, Foot Flat transition, and both the sensitive elements of the heel and the metatarsus show values other than zero. Immediately afterwards, there is an exchange of the forces exerted by the foot on the ground with the heel that comes off the ground and brings the $X_{h}\left(t\right)$ signal towards values close to zero and the metatarsus reaching its maximum value. At this moment, we are in the peak of the $X_{m}\left(t\right)$ signal (indicated with the dashed circle in Figure 6), which shows that the weight is fully loaded on the forefoot and that the foot is ready to leave the contact with the ground, ending the stance phase.

### 3.2. Step Count and Frequency Validation

To validate both algorithms for step count and the step-to-step frequency, we correlated (Pearson’s correlation) the automatic estimations with the reference obtained by manual annotation of step count and step-to-step time intervals for each trial.

**Figure 7:**
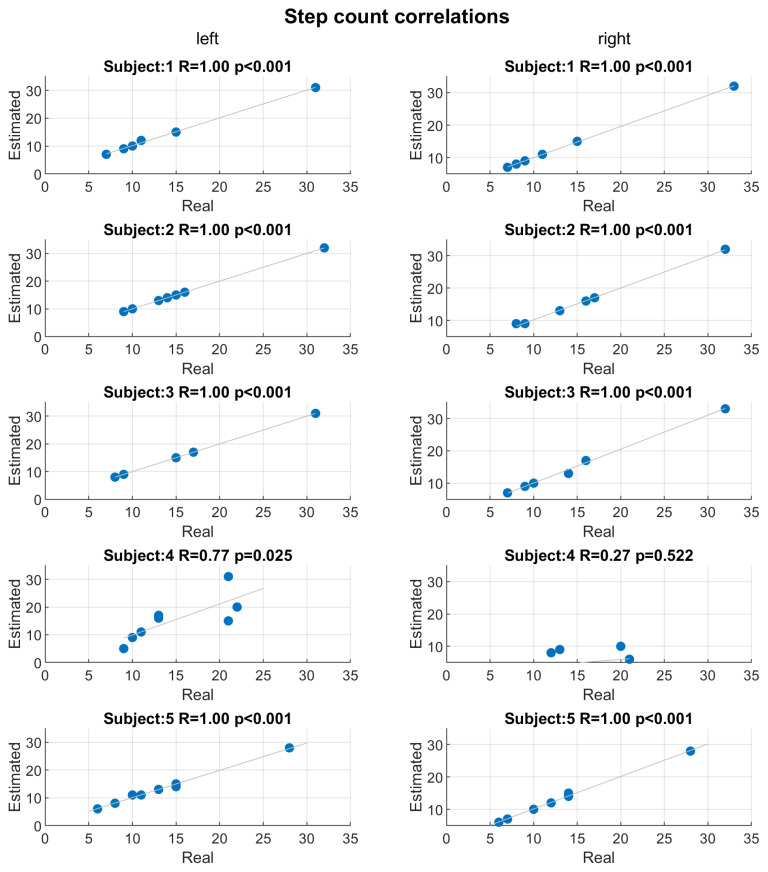
Scatter plots and Pearson’s correlations between step counts estimated by developed algorithm and real counts. Scatter plots are reported for each subject and correlation considering, separately, the left (**left column**) and right (**right column**) socks. For each correlation, the Pearson’s correlation coefficient (R) and corresponded *p*-value (*p*) are reported.

**Figure 8:**
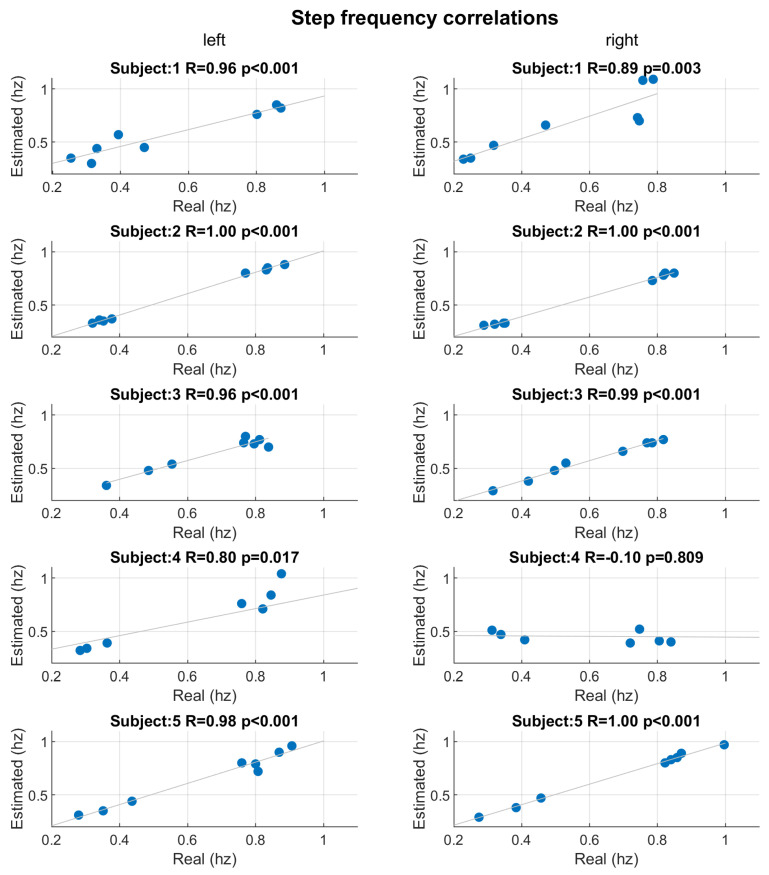
Scatter plots and Pearson’s correlations between mean step-to-step frequency estimated by developed algorithm and real frequency. Scatter plots are reported for each subject and correlation considering, separately, the left (**left column**) and right (**right column**) socks. For each correlation, the Pearson’s correlation coefficient (R) and corresponded *p*-value (*p*) were reported.

Figure 7 shows the scatter plots and Pearson’s correlations between step counts estimated by the developed algorithm and real counts for each subject and both socks. Except for subject 4, the correlations are extremely high: the correlation coefficients (R) are 1 and statistical significant (*p*-value <0.001). Only subject 4 shows low performance in step count estimation, in particular for right socks (R=0.27, *p*-value =0.522). For all the subject, except for subject 4, the maximum error between the step count estimation and real measure was ±1 step. Figure 8 shows the scatter plots and Pearson’s correlations between mean step-to-step frequency estimated by the developed algorithm and real mean frequency for each subject and both socks. As in the case of step count, except for subject 4, the correlations are significant (*p*-value ≤0.003) and with very high correlation coefficients (R≥0.89). The signals collected by subject 4 were highly artefacted and noisy due to being worn out.

The statistical comparison did not show significant differences between the results obtained from left and right socks for both step count and step-to-step frequency estimation (paired Wilcoxon Signed Rank Test, p=0.75 and 0.45, respectively).

The developed algorithms for step count detection and walking frequency estimation showed optimal performances in the case of non-artefacted signals from the sensing socks. In the case of step count, the results showed an almost perfect correlation (R=1) between real measure and the estimation. For the step-to-step frequency, the correlations with references remained very high (R≥0.89). Obviously, the results are strongly dependent on the quality of the collected signals. Furthermore, the developed algorithms also permit one to estimate the dynamic of step-to-step frequency, by proving an estimation of step frequency as a function of the time.

### 3.3. Study Limitations

In this work, we reported the realization of an innovative wearable system for gait analysis based on a sensorized sock that integrates a patch of matrix sensitive elements completely textile, flexible, and elastic. The preliminary results obtained are promising, but at the same time some limitations of our work must be mentioned. The principal aspects concern the tests carried out and the subjects involved. In fact, only healthy subjects with normal motor skills were selected, who were asked to walk with different gait modalities (speeds of execution of the test), which were obviously very different from the pathological gaits of a subject with specific motor or neurological deficits. However, our study was conceived to demonstrate the innovative prototype and to perform a preliminary evaluation of the technology made to provide easily processable signals, i.e., the use of the averages of the values of the sensitive elements, which, with appropriate algorithms, allow for the extraction of parameters related to the gait of the subject. Furthermore, we wanted to test the system during free walking, in those conditions that best represent the activities of everyday life. For this reason, we did not forsee the use of further laboratory equipment that could condition and/or limit the movement of the subject. With a view to future work, it will be necessary to develop a more intensive test phase that includes healthy and pathological subjects, and in which the gold standard instrumentation for gait analysis, such as force, inertial, and optoelectronic systems, is used for the generation of the reference signal used to compare and validate the parameters extracted from the sensorized sock. Under these conditions, it will be possible to evaluate more complex parameters of gait analysis, such as stride length and speed, and validate the reliability of our system in estimating these parameters. Furthermore, in future studies we plan to use the high number of sensitive elements integrated in our sensorized sock to also carry out static analysis of the subject’s posture, therefore evaluating the balance and the support modality of the entire plantar arch, in comparison to the analysis carried out by common podobarometric platforms.

## 4. Conclusions

In this paper, we have presented an innovative, low-cost wearable device based on a sensorized sock that is useful for long-term gait analysis such as during activities of daily living. Starting from the classic resistive matrix method, we have developed a sensing patch in which we have introduced many relevant improvements to overcome the limitations of the classic method. In particular, we have created the structure of the intermediate layer based on a piezoresistive fabric divided into single strips applied only in the area of interest to minimize the parasitic resistances typical of the resistive matrix method and to optimize the elasticity of the entire sensorized sock. In addition, we designed the sensing patch by distributing all 12 electrical contacts on the same layer in the ankle area to improve the adhesion of the electronic unit and the fit of the sensorized sock. We have developed two sensorized socks, one for the left foot and one for the right foot, which differ for the strategies implemented for connecting the sensing elements to the electronic unit. Compared to existing sensorized socks, the design described in this paper, based on the adaptation of the resistive matrix method, has enabled the possibility to obtain a fully stretchable prototype with an high number of sensors. We have developed dedicated algorithms for the extraction of the number of steps and step frequency that we have tested and validated on the samples obtained from laboratory tests. The results of the analysis of the 80 tests performed by 5 subjects, even if preliminary considering the low number of subjects tested and number of trials carried out, show an excellent correlation between the estimated parameters and the real ones, demonstrating how the sensorized sock can become a useful and reliable tool for gait analysis in everyday conditions. Future studies will aim to carry out trials on a larger number of subjects, allowing us to verify how much the sensorized sock is subject to wear during daily use, how many washing cycles it can withstand while maintaining the characteristics of the system, how sweat or other typical foot bacteria can influence the response of the sensors.

## 5. Patents

Concerning the fabrication of the prototype, we have filed an Italian patent application in the name of the University of Pisa.

## Figures and Tables

**Table 1:** Subjects Characteristics.

Age (Mean ± SD)	Sex	Height [cm] (Mean ± SD)	Weight [Kg] (Mean ± SD)	Foot Size (Mean ± SD)
25.6 ± 1.96	F	168.3 ± 6.6	62.6 ± 9.3	38.8 ± 1.7

## Data Availability

Data are available on request.
